# Tribulations of the Last Mile: Sides from a Regional Program

**DOI:** 10.3389/fvets.2017.00004

**Published:** 2017-01-31

**Authors:** Victor J. Del Rio Vilas, Mary J. Freire de Carvalho, Marco A. N. Vigilato, Felipe Rocha, Alexandra Vokaty, Julio A. Pompei, Baldomero Molina Flores, Natael Fenelon, Ottorino Cosivi

**Affiliations:** ^1^Pan American Foot-and-Mouth Disease Center (PANAFTOSA), Pan American Health Organization, Regional Office of the World Health Organization for the Americas (PAHO/WHO), Rio de Janeiro, Brazil; ^2^School of Veterinary Medicine, University of Surrey, Guildford, UK

**Keywords:** rabies, canine mediated, America, regional program, elimination

## Abstract

In Latin American and Caribbean (LAC) countries, the number of cases of dog-mediated human rabies is at its lowest since the onset of the Regional Program for Rabies Elimination in 1983, a commitment from LAC countries to eliminate dog-mediated rabies coordinated by the Pan American Health Organization. Despite minor setbacks, the decline in the number of human cases has been constant since 1983. While many LAC countries have significantly reduced rabies to a level where it is no longer significant public health concern, elimination has proven elusive and pockets of the disease remain across the region. In the 33-year period since 1983, the region has set and committed to four dates for elimination (1990, 2000, 2012, and 2015). In this paper, we ponder on the multiple causes behind the elusive goal of rabies elimination, such as blanket regional goals oblivious to the large heterogeneity in national rabies capacities. Looking ahead to the elimination of dog-mediated rabies in the region, now established for 2022, we also review the many challenges and questions that the region faces in the last mile of the epidemic. Given the advanced position of the Americas in the race toward elimination, our considerations could provide valuable knowledge to other regions pursuing elimination goals.

## Introduction

In 1983, when dog-mediated rabies in the American region was the cause of over 200 human deaths and 12,000 dog cases per year, representatives from the countries, gathered at the first Regional Meeting of Rabies Program Directors (REDIPRA) (1) coordinated by the Pan American Health Organization (PAHO/WHO), had the vision of a future free of dog-mediated rabies. Armed with nerve tissue vaccine for dogs, they launched a region-wide plan leading to mass dog vaccination campaigns across the region and set up the first elimination goal for the Americas, by 1990. Three other elimination goals followed in 2000, 2012, and 2015. Although the goal was not achieved by 2015, the coordinated regional efforts toward elimination led to the control of dog-mediated rabies in most of the Americas. At the time of writing, eight dog-mediated human rabies cases have been reported across the region in 2016, all in Haiti.

In the following, we ponder on the possible reasons that may have contributed to the four missed elimination goals to date, and specifically target the final years of the regional control program. We then speculate on the main challenges that lay ahead. Our considerations, necessarily from a regional perspective, are based on the experience of the region in the race toward elimination and could provide valuable knowledge to other regions pursuing similar goals.

## Challenges Past and Future

### On Program Management

At the announcement during the last REDIPRA meeting in 2015 (1) that the fourth elimination goal was not going to be achieved, PAHO and the countries did not establish another deadline, but agreed on a pathway toward the definition of the next goal. Two possible future goals were discussed: (i) elimination of canine rabies or (ii) elimination of dog-mediated human rabies. Countries chose the latter, as they did on the previous four occasions. Regardless of the scope of the goal, countries agreed to the recommendation that the new date for elimination had to be based on the systematic evaluation of countries’ rabies capabilities. This approach to goal setting differs from the previous four that led to arbitrary elimination dates that failed to recognize the heterogeneous development of rabies capabilities among countries.

Proactively coordinated, the aggregated evaluation of countries’ rabies capacities, and their improvement plans toward disease elimination should inform the earliest date at which the region would be able to eliminate the disease. Moreover, the aggregation of gaps from all countries would inform the regional demand for specific capacities, e.g., rabies vaccine, some of which could be more efficiently provided by a regional mechanism (e.g., PAHO’s Revolving Fund) (1).

The development of a systematic evaluation framework of the countries capacities is thus critical. Such a framework would require the definition of regional standards and indicators as well as clear requirements regarding the nature and quality of the evidence needed to support control and elimination claims. A PAHO review of indicators used by the countries to monitor the performance of their rabies programs identified large heterogeneity, in the number of indicators per country, from just a few to more than 100, and in the nature of them, from process indicators to outcome indicators (2).

The importance of the regular REDIPRA meetings, which constitute the strategic governance platform of rabies programs in the region, cannot be underestimated. Group dynamics prevalent at these meetings exercise great influence on strategic issues that cannot be replicated remotely. Specifically, we stress the importance of peer-pressure among countries. In addition, regional coordination requires formal structures to facilitate regular networking in the interval between REDIPRA meetings, e.g., via working groups around specific products such as interlaboratory proficiency exercises (1).

On coordination, we must mention stakeholder engagement, even if briefly, and specifically one of the most salient actors in recent years, i.e., animal rights groups. The engagement of officials with these groups was not always productive and, at times, led to departures from the real focus on rabies control. But a change at both camps appears evident in recent years. Both sides have learned to moderate their message, and now understand that negotiation and not confrontation leads to better outcomes for all. Official programs start to recognize that these groups need to be brought to the discussion table at the planning stages, as they can deliver niche-specific approaches to local problems.

### On Evidence

The impact of interventions can only be monitored with reliable data. Since 1998, PAHO has been collecting data via questionnaires to the countries on their programs’ performance prior to the regular REDIPRA meetings. It was only at the most recent REDIPRA in 2015 that a thorough analysis of the data could be presented (1).

Earlier reports stated that a number of Latin American and Caribbean (LAC) countries were conducting excellent rabies surveillance (3). These results seem to concur with those reported by zoonosis managers to a survey in early 2015 who replied that they were satisfied with the sensitivity of their rabies surveillance (4). However, to the best of our knowledge, and with the exception of Haiti (5), there is no systematic evaluation of rabies surveillance in the region, and we are not aware of recent patient chart reviews of acute encephalitis or sensitivity estimation of dog surveillance. Specifically on the latter, the region, by large, follows the recommendation of sampling a proportion of the dog population (3). This, at best, has facilitated discussions on the importance of targeting dogs for early warning and, at worst, has drained resources without informing the epidemiology of the disease. Recent work supported by PAHO questions this approach to surveillance and suggests more efficient alternatives (6). These studies also show the importance of variant identification, especially at the end game, and are a reminder that some countries in the region still lack this capacity in-house, or even nimble mechanisms to acquire it elsewhere for prompt response to cases.

The investigations that followed the recent occurrence of multiple dog cases in Brazil (7), spanning to more than one local authority, highlighted fundamental structural problems, not rabies-specific, for the generation of sound evidence, i.e., the absence of a common standard for data gathering across administrative units. This is likely to resonate in other countries, and it highlights the need for a standard epidemiological report. To that effect, the region will benefit from the ongoing efforts by Brazil toward the harmonization of processes across its network of zoonosis’ surveillance units.

The regional rabies database, SIRVERA (8), despite all its shortcomings, has played a critical role in the success of the regional program. A perhaps overlooked contribution is that SIRVERA is the most tangible product of the program across its many years and participating countries. Together with other “brands” of the program (e.g., REDIPRA), SIRVERA has bundled the countries around the regional goal. That is, those countries that contribute data to it. Three countries where rabies is still endemic have consistently failed to report to SIRVERA. Such failure to report not only impacts on the ability of the program to monitor regional progress, but it has important consequences on neighboring areas pursuing control and elimination as they struggle to assess the risk of incursion from such countries. Like the other capacities, SIRVERA needs to change to adapt to the end game too and become the exhaustive repository of rabies programs performance indicators in the region. In other words, it must not just chase cases but also track and identify substandard capacity planning and deployment that constitute the best early warning of rabies risk.

### On Resources

A prolonged epidemic tail, consecutive goal failures and farther in time goals, contribute to investors’ fatigue (in the case of the Americas, these are mainly government budget holders, with external donors playing a much limited role, except for Haiti). It is a well-described fact that short-term goals lead to greater willingness to invest (9). The opposite can lead to reduced donor engagement. Efforts to attract resources must contemplate breaking down long-term regional goals, to reduce the long payback period, into country and area-specific objectives with short- and medium-term deliverables linked to enhanced capacity deployment to attract more investors/donors seeking quick returns. In other words, investment opportunities need to be indexed to processes and capacities that are fully measurable, tractable, and prone to direct influence. Moreover, short-term successes, e.g., the declaration of an increasing number of rabies-free countries and areas along the way, would reduce the perception of uncertainty around the overall investment for regional elimination, farther ahead.

Efforts to attract investment must also recognize that at the end game, there is little room for inefficiencies. As a result, optimization of regional and national resource allocation schemes, e.g., by country or geographical area vs. by capacity, merits study to prevent underperforming assets from receiving undeserved support (10, 11). This tendency may stem from failure to accept underperforming capacities relative to others, or a lack of appreciation of the full scope of opportunity costs. This might well be the case of devoting scarce resources to dog sterilization, an intervention that delivers a much lesser punch at a much greater cost than dog vaccination. Inefficient investment will only prolong the tail end of the epidemic, directly resulting in further cases and unnecessary deaths and, as a result, increasing the risk of goal fatigue. Other inefficiencies, either at the national or regional level, could occur as a result of maintaining vaccine production facilities for reduced domestic demand, unsubstantiated dog surveillance strategies that lead to no useful evidence for decision making, or the current prophylaxis schemes (PEP), promoting intramuscular administration, prevalent across the region.

### On Vaccination

Notwithstanding occasional problems, all countries in the region, except for those that have been free from rabies for years, plan the purchase of vaccine (for humans and dogs) in their annual budgets. This, in comparison with other regions, is a feast. Not all, though, manage to acquire the vaccine at all (most notably Haiti), or in the quantities and timelines needed. The insufficient deployment of vaccine, whether in control or elimination stages, is the ultimate reason as to why rabies persists in some of those countries. Insufficient dog vaccine deployment, due to deficient population coverage, untested vaccine quality, mismanagement of batches, and non-compliance with protocol, translates into failure to consistently achieve herd immunity in a number of scattered locations that remain endemic, even if undetected. Occasional donations and exchanges of vaccines between countries, whether brokered by PAHO or not, have been the norm in the past to supply vaccine to areas in acute need (3, 12). This solidarity may be threatened as more areas reach elimination in the region, and hence reduce the size of their rabies programs and vaccine stockpiles. In such a scenario, where one single exposure may delay the region’s goal, a regional mechanism to guarantee rapid deployment of rabies vaccines merits consideration.

Following on our remarks about the limited appetite for inefficiencies at the last mile, dog vaccine application must seek ways to reduce repeated vaccination of easy-to-reach animals. Beyond the financial implications, these animals contribute to vaccine coverage indicators, despite bringing no additional immunity, and may lead to a false sense of achievement about herd protection in the targeted dog population.

Rabies programs across the region have benefited from the incorporation of human rabies vaccine in the countries’ regular acquisition of biologicals through PAHO’s purchasing mechanisms. In 2014, following recommendations from REDIPRA (13), PAHO also included the dog vaccine in its portfolio of biologicals on offer through the PAHO revolving fund. Via large purchases, PAHO guarantees the provision of quality vaccine at competitive prices, and, most importantly, promotes regular budgeting practices in the countries. Improvements are possible, for example, by incorporating human rabies vaccine in the well-established logistical systems of the Expanded Programme on Immunization in country. This is a work in progress after a communication by PAHO to that effect was sent to the countries last year.

Despite seeing the lowest human case count in over 30 years, the demand for human rabies vaccine is on the rise. If human vaccine sold by PAHO’s purchasing mechanisms is a good proxy for the overall use across the region, bearing in mind that not all the LAC countries buy through PAHO, we have noted an average increase of over 55,000 doses of human vaccine every year for the period 2005–2015. In 2015 alone, circa of 900,000 doses were acquired via PAHO’s revolving fund. Although alternative dog surveillance systems, such as those based on tracking exposures after reported bites, could lead to more efficient PEP application (6), rabies programs must prepare for long-lasting demand of PEP even in the absence of canine rabies cases for quite some time.

### On Awareness

In contrast to the evident scars left by smallpox, or other diseases with obvious sequelae, rabies does not leave living bearers to remind us of its occurrence. Together with the declining incidence, awareness of the disease will wane. This phenomenon will only get worse, and it would be important to regularly monitor the levels of awareness among the population in risk areas. Activities to that effect, e.g., knowledge-attitudes and practices surveys, may deliver collateral benefits by capturing heightened risk perception among the population that may provide sufficient reason, in the absence of adequate surveillance, for policy intervention.

A decrease in awareness may also lead to reduced uptake of preventive measures as seen in other diseases, e.g., measles vaccine. For rabies, the impact might be twofold: leading to reduced dog vaccination, and PEP uptake and prescription by health staff after exposure. The latter was the target of the recent rabies alerts issued by PAHO after a number of cases in the region (14). However, no similar alerts were issued after evidence of insufficient dog rabies vaccines coverage in risk areas. This, again, highlights the reactive nature of the program chasing cases and not capacities (or their absence).

## Final Remarks

It has been said that the regional rabies elimination program is a victim of its own success, as reduced disease incidence leads to relaxation of controls, and new evidence needs at the end game challenge attitudes and practices that worked well during the control phase, but may not do so well during the last mile. Given the regional success, some resistance to accept innovations tested in other settings, as is the case of intradermal human rabies vaccine or the use of capture–recapture methods to estimate dog vaccine coverage, is expected. Transferring successful approaches from well-controlled projects in local settings elsewhere is not without difficulty as managers tend to dismiss them as generated from different contexts of little applicability to their own. This may be true, given well-known limitations in external validity of even the most robust investigations (15). However, the merit of these well-controlled studies elsewhere is undeniable, and the region is now benefiting from such findings in the formulation of canine surveillance guidance and area level classification.

Basic problem structuring theory identifies two types of complexities in every problem and decision setting to its resolution: technical or analytical complexities, and organizational complexities (16). For rabies in the Americas, where effective tools are available for its control and elimination, disease persistence is due to organizational failure at the planning, implementation, or evaluation of the rabies program. Specifically, failure to (i) gather and properly present the evidence about disease risk and vulnerabilities to budget holders, (ii) systematically generate synergies with other programs and stakeholders to ensure efficient capacity deployment, and (iii) conduct thorough risk assessment on the sustainability of the rabies program. We recognize the impact of externalities on any disease program, but for dog rabies and the Americas, the authors believe that contextual factors play a critical role in one country alone: Haiti.

Improvements are underway. The regional program has now developed a framework for evaluation of rabies capacities, commissioned research to robustly guide dog surveillance requirements (5) and classify areas based on a composite measure of risk (17), and recently released a new SIRVERA platform capable of managing all the evidence needs in the elimination phase (8). These tools come with a price. They need increasing amounts of quality and timely data to provide the required precision, around results of interventions such as dog vaccine coverage, to support robust and opportune decision-making.

Reaching out to countries to promote reporting as per the new data standards and to support the shift in focus toward monitoring capacities and vulnerabilities, and not just cases, will require increased resources by the regional program. The additional resource will have to be distributed across endemic, at risk and free areas. Although the most obvious targets are the endemic and at risk classes, the region must also capitalize on those areas that achieve freedom. To that effect, the regional program needs to formalize the processes around rabies elimination and maintenance of such status and demand thorough risk analysis by countries that contemplate interventions beyond their borders in collaboration with areas still posing a risk.

Good as a planned approach may be, elimination requires more than cold preparation. Failure to reach the 2015 elimination goal should have generated a state of crisis to justify major transformational changes in the regional program. Without such changes, in the form of a state of urgency to propel the commitment to prompt elimination, the risk of apathy is real. Without consideration of the intangible benefits stemming from elimination, mostly of a political nature, the cold calculations around the diminishing returns of additional disease control measures at this stage may lead to the perpetuation of the current situation. This would betray the vision of those colleagues over 30 years ago. Without the pressure of an imminent goal, there are no regional consequences as a result of a new case, beyond the tragic death of a human being by a shameful disease.

The recently approved “Plan of Action for the elimination of neglected infectious diseases and post-elimination actions 2016–2022” (18), should deliver the new impetus, and the resources, to reach rabies elimination by 2022. Of mention is the inclusion in this plan, for the first time, of a reference to the elimination of canine rabies transmission.

## Author Contributions

VV conceived and wrote the work. All the authors have critically reviewed and revised the manuscript and approved it for publication.

## Conflict of Interest Statement

The authors declare that the research was conducted in the absence of any commercial or financial relationships that could be construed as a potential conflict of interest. The handling editor declared a past co-authorship with one of the authors MV and states that the process nevertheless met the standards of a fair and objective review.
